# Urology consultants versus large language models: Potentials and hazards for medical advice in urology

**DOI:** 10.1002/bco2.359

**Published:** 2024-04-03

**Authors:** Johanna Eckrich, Jörg Ellinger, Alexander Cox, Johannes Stein, Manuel Ritter, Andrew Blaikie, Sebastian Kuhn, Christoph Raphael Buhr

**Affiliations:** ^1^ Department of Urology University Hospital Bonn Bonn Germany; ^2^ School of Medicine University of St Andrews St Andrews UK; ^3^ Institute of Digital Medicine Philipps‐University Marburg and University Hospital of Giessen and Marburg Marburg Germany; ^4^ Department of Otorhinolaryngology University Medical Center of the Johannes Gutenberg‐University Mainz Mainz Germany

**Keywords:** artificial intelligence (AI), Bard, chatbots, ChatGPT, digital health, global health, large language models (LLMs), low‐ and middle‐income countries (LMICs), telehealth, telemedicine, urology

## Abstract

**Background:**

Current interest surrounding large language models (LLMs) will lead to an increase in their use for medical advice. Although LLMs offer huge potential, they also pose potential misinformation hazards.

**Objective:**

This study evaluates three LLMs answering urology‐themed clinical case‐based questions by comparing the quality of answers to those provided by urology consultants.

**Methods:**

Forty‐five case‐based questions were answered by consultants and LLMs (ChatGPT 3.5, ChatGPT 4, Bard). Answers were blindly rated using a six‐step Likert scale by four consultants in the categories: ‘medical adequacy’, ‘conciseness’, ‘coherence’ and ‘comprehensibility’. Possible misinformation hazards were identified; a modified Turing test was included, and the character count was matched.

**Results:**

Higher ratings in every category were recorded for the consultants. LLMs' overall performance in language‐focused categories (coherence and comprehensibility) was relatively high. Medical adequacy was significantly poorer compared with the consultants. Possible misinformation hazards were identified in 2.8% to 18.9% of answers generated by LLMs compared with <1% of consultant's answers. Poorer conciseness rates and a higher character count were provided by LLMs. Among individual LLMs, ChatGPT 4 performed best in medical accuracy (*p* < 0.0001) and coherence (*p* = 0.001), whereas Bard received the lowest scores. Generated responses were accurately associated with their source with 98% accuracy in LLMs and 99% with consultants.

**Conclusions:**

The quality of consultant answers was superior to LLMs in all categories. High semantic scores for LLM answers were found; however, the lack of medical accuracy led to potential misinformation hazards from LLM ‘consultations’. Further investigations are necessary for new generations.

## 1 INTRODUCTION

‘I searched my symptoms on the internet…’ is a frequently made statement by patients consulting medical advice in hospitals and private practice alike these days. This prevalent behaviour is a direct result of the ubiquitous availability of medical information from the internet. Although an informed patient is a desirable scenario, misinformation and wrongful preconceptions may hinder a trusting relationship with the attending healthcare professional.^1^, ^2^

The current hype surrounding AI and large language models (LLMs) will also transform the process of medical self‐information. Internet research formerly conducted through search engines like Google will transfer to open access services like Bard and ChatGPT (Chat‐Generative Pre‐trained Transformer), which are now being used for a broad range of tasks including medical information queries. Hence, LLM services will likely soon replace the traditional search engines as the primary source of medical information for lay people.^3^, ^4^, ^5^, ^6^

LLMs are powered by a ‘deep neural network architecture’ and leverage principles of natural language processing. These models employ unsupervised learning, where they analyse extensive text data to learn linguistic patterns, grammar rules and contextual nuances. Deep neural networks, composed of multiple layers of interconnected nodes, process sequential data, such as sentences, by predicting the next word based on preceding words, developing a profound understanding of language structure. Hence, they excel in tasks like text generation and demonstrate remarkable linguistic abilities. LLMs are continuously trained on a vast amount of data including books, articles, websites and other textual content. The increasing amount of training data is just one factor contributing to continual model improvements. Architecture enhancements, fine‐tuning techniques and improvements in training algorithms continue to progress and enhance the capabilities of these models.^7^

Beyond LLMs' vast database, their output of apparently human‐like answers, as well as their multilingualism, makes them a seemingly competent contact point for medical consultation. However, using LLMs as a substitute for medical caregivers bears significant risks. The flawless semantic form may drive patients to avoid professional advice and lead to wrong diagnostic or therapeutic conclusions. These could result in severe misinterpretation of symptoms, hypochondria, increased anxiety and potentially harmful self‐treatment or non‐treatment.^1^, ^8^ The motivation behind internet research of medical symptoms is versatile and ranges from limited access to healthcare to the desire for reassurance or a second opinion. Furthermore, the search for deeper understanding and perceived external barriers to accessing information through traditional sources play a significant role. Additionally, factors like convenience, coverage and anonymity of medical internet research are relevant in this regard.^9^

Embarrassment, cultural ostracism and rejection of conventional norms emerge as pertinent determinants contributing to the avoidance of seeking counsel from medical practitioners, especially in medical domains such as urology. However, this delay leads to possibly adversarial postponement in the instigation of medical intervention.^10^, ^11^ This emphasizes the relevance of medical internet research, particularly in contexts encompassing potentially awkward or humiliating medical conditions.

Previous studies showcased by our working group showed the general ability of LLMs to answer medical case‐based questions in the field of otorhinolaryngology correctly. Yet, the overall medical adequacy of the answers given was significantly inferior to those of specialists given the same questions.^3^ As we believe in the high relevance of this topic, to further evaluate the capabilities and limitations of LLMs as providers of medical advice, we compared the answers given by three different LLMs for case‐based medical questions to answers provided by specialists in the field of urology.

## 2 METHODS

Urology study books, exemplary questions from urological journals and former exams were browsed for case‐based questions. The selected questions were matched to clinical cases from the outpatient unit and the emergency centre, and corresponding questions were selected.^12^, ^13^

After this process, 45 questions were selected resembling a broad range of urological pathologies. Subsequently, the questions were answered by four urology consultants (co‐authors of this article) and three selected LLMs, respectively. LLMs ChatGPT 3.5 (free version of ChatGPT during trial), ChatGPT 4.0 (latest [paid] version ChatGPT) and Google Bard were utilized for this study because of their broad use and low barrier setup. On the other side, the consultants selected had at least 6 years of clinical experience in urology.

After the questions were answered, they were again randomized. A character count for every answer was determined and statistically compared.

After randomization, all answers given by the urology consultants and the LLMs respectively were rated by the homologue 4 consultants (their own questions excluded) using a 6‐point Likert scale (1 = very poor, 6 = excellent). It must be noticed that answers provided by LLMs often include phrases that disclose a lack of qualification to answer medical questions or advise a medical consultation. To avoid possible bias and to allow a modified Turing test, these phrases were excluded before further evaluation.

Questions were rated for medical adequacy, conciseness, coherence and comprehensibility respectively in concordance with previous studies by our working group.^3^

Additionally, the hazardous potential of the answers provided was rated in a binary rating system (possible hazard: yes/no).

With the corresponding rating operandus, the consultants also assessed whether the answers were created by a urology consultant or by one of the LLMs respectively.

Gaussian distribution of ratings was evaluated after data acquisition utilizing the D'Agostino & Pearson test. After normality testing was performed, pairwise comparisons were realized with the non‐parametric Mann–Whitney test or the Kruskal–Wallis test if more than two groups were compared. The statistical testing was performed using GraphPad Prism, Version 10.0.3 (GraphPad Software, La Jolla, California, USA).

## 3 RESULTS

**FIGURE 1:**
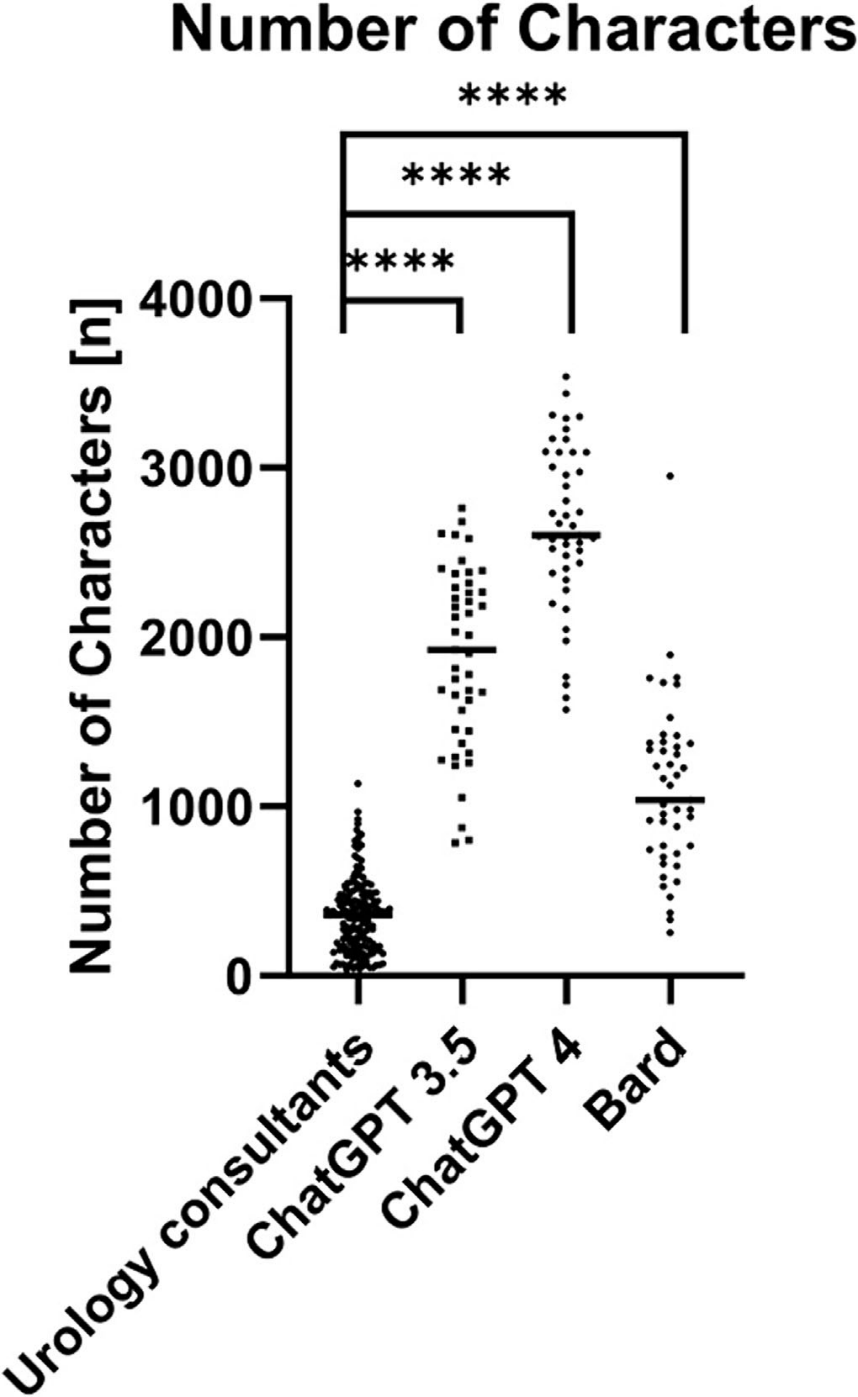
The number of characters per answer by urology consultants and large language models (LLMs; ChatGPT 3.5, Chat GT 4, Bard) for all evaluated categories. Data shown as a scatter dot blot with each point resembling an absolute value. Grey horizontal line = Median. The non‐parametric Mann–Whitney test was used to compare the ratings for individual LLMs to the urology consultants (**** = *p* < 0.0001).

As shown in Figure 1, evaluation of the character count showed a significantly reduced semantic output of the consultants compared with all three LLMs. Although the urology consultants utilized a Median of 359 characters per answer (Range 39–1135), ChatGPT 3.5 used 1926 (786–2762), ChatGPT 4 2600 (1572–3537) and Bard 1039 (256–2951) characters.

Even though the semantic quality of answers was rated comparatively good (Table 1), answers by the urology consultants were correctly assigned as the source of the origin for 99.01%, whereas the LLM was identified correctly in 98.00% of cases.

**TABLE 1:** Cumulative ratings for all categories evaluated by the urology consultants.

	Ratings (*n*)	Rating (Median; [Range])	Rating (Mean)	Rating [95% CI]	*p*‐value^a^	*p*‐value^b^
Medical adequacy						
Urology consultants	540	6 (1–6)	5.687	[6;6]		
ChatGPT 3.5	180	5 (1–6)	4.661	[5;5]	<0.0001	<0.0001
ChatGPT 4	180	5 (2–6)	5.200	[5;6]	<0.0001	
Bard	180	5 (1–6)	4.244	[4;5]	<0.0001	
Conciseness						
Urology consultants	540	6 (3–6)	5.893	[6;6]		
ChatGPT 3.5	180	5 (1–6)	4.450	[4;5]	<0.0001	n.s.
ChatGPT 4	180	5 (2–6)	4.444	[5;5]	<0.0001	
Bard	180	5 (1–6)	4.350	[4;5]	<0.0001	
Coherence						
Urology consultants	540	6 (4–6)	5.765	[6;6]		
ChatGPT 3.5	180	5 (4–6)	5.500	[6;6]	<0.0001	=0.001
ChatGPT 4	180	5 (2–6)	5.611	[6;6]	=0.0052	
Bard	180	5 (4–6)	5.289	[5;6]	<0.0001	
Comprehensibility						
Urology consultants	540	6 (4–6)	5.785	[6;6]		
ChatGPT 3.5	180	6 (4–6)	5.561	[6;6]	<0.0001	n.s.
ChatGPT 4	180	6 (4–6)	5.678	[6;6]	=0.0234	
Bard	180	6 (3–6)	5.561	[6;6]	<0.0001	

*Note*: Comparative statistics between the specific large language model (LLM) and the consultants were carried out using the Mann–Whitney test. A comparative statistic evaluation between the three LLMs was carried out using the Kruskal–Wallis test.

^a^ Compared to ratings of the urology consultants with the Mann–Whitney test.

^b^ Comparison between the three different LLMs with the Kruskal–Wallis test.

**FIGURE 2:**
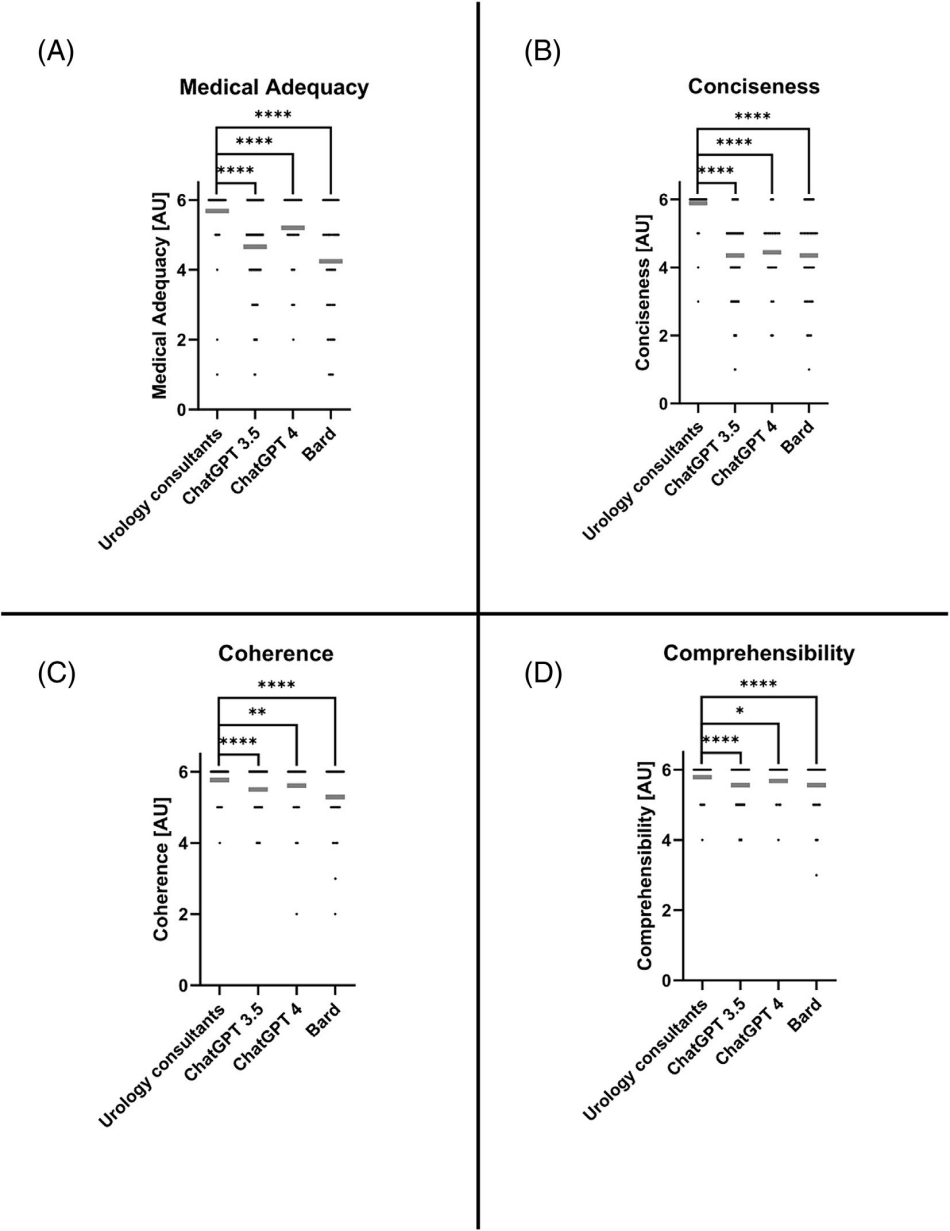
Comparison between urology consultants and large language model (LLMs; ChatGPT 3.5, Chat GPT 4, Bard) for all evaluated categories. Data shown as a scatter dot blot with each point resembling an absolute value. Grey horizontal line = Median. The non‐parametric Mann–Whitney test was used to compare the ratings for individual LLMs to the urology consultants (**** = *p* < 0.0001; ** = *p* < 0.01; * = *p* < 0.05). Cumulative results of ratings for medical adequacy (A), conciseness (B), coherence (C) and comprehensibility (D).

As shown in Figure 2, answers provided by urology consultants were rated superior to answers provided by the LLMs in every category.

A more detailed depiction of individual ratings and proportions is provided in Table 1.

Particularly, the ratings for medical adequacy and the conciseness of answers provided showed a relatively high qualitative discrepancy between urology consultants and the LLMs (*p* < 0.0001). Although differences in the rated categories still reached statistical significance, LLMs' performance was noticeably better in semantic evaluation criteria (coherence [*p* = 0.0052–*p* < 0.0001] and comprehensibility [*p* = 0.023–*p* < 0.0001]).

We noticed significant differences between the individual LLMs regarding the ratings for medical accuracy (*p* < 0.0001) as well as coherence (*p* = 0.001). In both categories, ChatGPT 4 was rated the most proficient, whereas Bard was rated with the lowest scores in both categories. Ratings for conciseness and comprehensibility, however, did not show any significant differences between all three LLMs.

To assess whether the answers provided could be the source of possible hazard, a binary rating system was included. Of the answers provided by urology consultants, 0.56% were rated as possibly hazardous for the patient, whereas answers provided by LLMs were rated as possibly hazardous in 2.78% for ChatGPT 4, 8.33% for ChatGPT 3.5 and 18.89% for answers provided by Bard. These findings are consistent with the distribution of ratings for medical adequacy for the individual LLMs.

Urology consultants were able to determine the source of the answers correctly in 99.01% for urology consultants and 98.00% for LLMs. For sample questions and answers, see Data S1.

## 4 DISCUSSION

The potential of LLMs is a heavily discussed topic in today's society—and for a good reason! LLMs now offer the possibility of access to medical information in a convenient and understandable way. They are therefore very likely to be used by patients as a source of medical information. Hence, evaluation of their potential as well as their limitations is important. The quality of output as well as the accuracy of responses must ultimately be critically re‐evaluated especially in the field of medical care.^14^, ^15^, ^16^

Our study therefore evaluated the performance of three commonly used LLMs for answering case‐based questions in the field of urology. As expected, the LLMs' responses were of high semantic quality as underlined by the high‐ranked overall comprehensibility and coherence (Table 1 and Figure 2). These findings support data recently published by Cocci et al. attesting a college graduate reading level for answers provided by ChatGPT as well as previously published findings by our working group in the field of otorhinolaryngology.^3^, ^17^ Nevertheless, even in semantic categories, the LLMs were still outperformed by the urology consultants as illustrated by their significantly higher ratings in the corresponding categories. Contrary to the relatively high comprehensibility and coherence of the answers provided, the LLMs showed a relative discrepancy regarding the conciseness of their answers compared with the urology consultants (Table 1/Figure 2). Taking these findings into consideration, it is important to acknowledge that the answers generated by LLMs were between 4 (Bard) to 9 times longer (ChatGPT 4) than the corresponding answers by the urology consultants (Figure 1). The lavish vocabulary, in contrast to the reduced conciseness, likely originates from the way that LLM chatbots are trained mimicking a ‘human‐like manner’ by using a rather complex speech pattern instead of stenographic language. According to the OpenAI website, this phenomenon traces back to the feedback of the testers, who preferred ‘longer answers that look more comprehensive’ [openai.com].^18^

Even though the LLMs achieved a high semantic quality in our rating, the urology consultants were able to determine the source of the answers correctly in 99.01% for urology consultants and 98.00% for LLMs, respectively. These findings may contradict the excellent performance of today's LLMs in the modified Turing test but could be heavily biased by the fact that an expert is rating answers in the field of personal expertise as well as by the repetitive semantic structure and the significantly longer answers.^19^

The medical adequacy of the answers provided is ultimately by far the most relevant criterion of evaluation. In this category, all LLMs were highly significantly outperformed by the urology consultants (Table 1/Figure 2). Although a Median adequacy of 5.0 for all LLMs still deserves credit for an entity not specialized in medical care, the poor performance is highlighted by the percentage of possible hazards. The latter ranges from 2.78% for misinformation responses for ChatGPT 4 and 8.33% for ChatGPT 3.5 up to 18.89% for answers provided by Bard.

Medical adequacy in this current study was however still rated higher than corresponding ratings in studies performed by other working groups.^17^, ^20^, ^21^ This difference may be accounted by the constant performance improvements of LLMs although the risk of misinformation still remains.

However, the potential of LLMs should not be ignored. In other specialties, LLMs have shown their potential to even outperform medical personnel as demonstrated by a recent study by Ayers et al.^22^ The authors evaluated ChatGPTs' potential in answering patient questions in comparison with a licensed physician. To assess the quality, answers to questions posted to a public forum were answered by a physician and chatbot alike and subsequently evaluated by healthcare professionals. Surprisingly, the trained personnel preferred chatbot responses to physician responses in 78.6% of the evaluations, even rating categories like empathy in favour of the LLM.

In our data, comparative analysis ratings for medical accuracy differed between the LLMs with ChatGPT4 being the most proficient of the three. Based on the limited sampling in our study, predictions on the evolution of medical accuracy of LLMs are hard to make. Yet, the significant increase in rating between ChatGPT 3.5 and ChatGPT 4 (*p* < 0.0001) may suggest improvement in that respect thereby contradicting recent findings by Zhu et al. and supporting findings by our own working group in the field of otorhinolaryngology.^3^, ^23^

As LLMs provide an accessible and well‐structured source of information, there are a variety of different use cases for LLMs in medical practice, ranging from providing additional information before consulting a doctor to low‐resource settings where a medical consultation in person is not available. Especially in urology, patients may find themselves in embarrassing situations, which is why they may want to avoid personal contact with the doctor. Other scenarios might occur after a diagnosis has already been made: the patient wants to gather more detailed information about their illness. In this manner, the LLM consultation can augment consultations with doctors and lead to empowerment of the patient for shared decision‐making.^24^

Furthermore, LLMs have the potential to complement health care leading to more cost‐efficient and timely delivery. Possible areas of applications include classification, organization and summarization of complex patient data, surveillance of complex medical co‐founders or management of the increasing bureaucracy in the healthcare system.^25^

Apart from the influence on the doctor‐patient relationship and economic effects, LLMs can improve global health issues in low‐ and middle‐income countries, especially in areas with limited and untrained staff. As smartphones and internet access are often available in these settings, LLMs may provide useful access to medical advice for immediate management and triage.

However, before the actual impact of LLMs in medicine on a wider scale can be implemented, there are still concerns to manage. LLMs specially trained for medical purposes, such as Med‐PaLM, will further improve the response to medical queries.^26^ Moreover, LLMs with real‐time access to the internet searching for up‐to‐date information and studies will take LLMs to the next level. Last but not least, special prompts will also optimize answers on medical questions. Here, our work can be helpful, as it reveals the inadequacies of the answers from a physician's perspective. Future work should analyse the needs and expectations of patients in more detail. Based on this information, further studies should build and evaluate LLMs with medical prompts on a larger scale in the future.

Whereas the lack of medical adequacy will likely improve when LLMs are specially trained for medical purposes, the regulation of LLMs handling highly sensitive patient and medical data might be more challenging and require strictly regulated and transparent standards.^27^, ^28^, ^29^ Potential risks for patients' privacy are highlighted by current legislative initiatives such as the EU Artificial Intelligence Act.^30^

Currently, there are two main approaches for dealing with potential privacy risks. First, it is the users' responsibility to consider carefully which data they are passing to the LLMs. Therefore, data should only be entered pseudonymized; moreover, using vpn clients can help to make it more difficult to assign the data to specific patients. Second, commercial providers such as Aleph Alpha already recognized the need for privacy protection offering an AI infrastructure where the rights on personal data remain entirely with the user.^31^ Unfortunately, these services have so far been exclusively reserved for commercial customers and are therefore only accessible for clinics, healthcare companies and authorities.

Obviously, our study has some limitations as only 45 case‐based questions were used as input instead of patients passing their symptoms themselves to the LLM. However, the provided rating by clinically experienced doctors represents the gold standard of medical care as a benchmark. Further studies should include real patients and proof of the performance of LLMs in urology on a larger scale.

Although our data accentuate the potential of LLMs regarding linguistic performance, the limited medical adequacy and the higher risk of misinformation hazard emphasize the jeopardy associated with an unsupervised use of LLMs as a source of medical information. Hence, we sincerely believe that LLMs should be considered as an augmentative tool for providing as well as seeking healthcare and not an autarchic entity.

## AUTHOR CONTRIBUTIONS

J Eckrich and CR Buhr conceived of the presented idea. J Eckrich browsed the textbooks and looked into the case based questions, which were then filtered and answered by J Ellinger, A Cox, J Eckrich and J Stein. CR Buhr entered all case based questions into the three LLMs, anonymized all answers. J Ellinger, A Cox, J Eckrich and J Stein rated the answers each by the other consultants and the LLMs. J Eckrich and CR Buhr then analyzed all answers and did the statistical evaluation. All authors discussed the results and contributed to the final manuscript.

## CONFLICT OF INTEREST STATEMENT

No third‐party funding was utilized for the design of the study and collection, analysis and interpretation of data and in writing the manuscript. The authors declare no competing interests.

## ETHICS STATEMENT

Written correspondence of March 3rd 2023 with the ethics committee of the regional medical association Rhineland‐Palatinate determined no need for any specific ethical approval due to the use of anonymous text‐based questions.

## Supporting information


**Data S1.** Supplementary Information.
