# Supplementary material for: Urology consultants versus large language models: Potentials and hazards for medical advice in urology
Source: BJUI Compass. 2024 Apr 3;5(5):438–44. doi: 10.1002/bco2.359 (PMC11090772; doi:10.1002/bco2.359)
Supplement: Supplementary file 1 — Data S1. Supplementary Information. [file BCO2-5-438-s001.docx]

**Supplementary S1**

All cases and questions were translated to English using deepl.com
(Cologne, Germany).

**Case 1:**

A 55-year-old patient has a renal cell carcinoma, which appears on CT as a 15 cm large mass at the lower renal pole with an additional renal vein thrombus. There are no detectable organ metastases, individual hilar lymph nodes appear enlarged. What are the treatment options?

**Urologist 1:**

Primarily, a CT thorax should be performed for thoracic filiarization. Due to the size of the tumor (at least pT2 stage) and the presence of a venous thrombus, there is an indication for nephrectomy, which can be performed open or laparoscopically depending on the size and length of the venous thrombus. As part of this, a lymphadenectomy should be performed for cN+. If the diagnosis of lymphogenous metastasis is confirmed (pN+) or a high-risk tumor is conclusively present (T2+G4, T3 or T4), adjuvant immunotherapy with pembrolizumab can be given for 12 months.

**Urologist 2:**

Nephrectomy with regional lymphadenectomy is recommended when there is evidence of a locally advanced renal tumor with questionable lymphogenic metastasis. The approach depends on the extent of the tumor thrombus in the renal vein; depending on the CT image, an MRI / sonography may also be necessary. In principle, a laparoscopic procedure is conceivable for smaller tumor thrombi in the renal vein, alternatively an open nephrectomy. If a locally advanced clear cell renal cell carcinoma is confirmed postoperatively (N+ or at least T3), the patient should be recommended adjuvant therapy with pembrolizumab for 12 months. Neoadjuvant drug treatment is not indicated apart from trials.

**Urologist 3:**

The patient is diagnosed with T3 renal cell carcinoma with local lymphogenic metastasis. MRI should be added to the imaging to evaluate the exact spread of the renal vein thrombus. Depending on the findings, an open or laparoscopic nephrectomy with lymphadenectomy can then be performed. Depending on the histopathological findings, adjuvant therapy may then be indicated.

**Urologist 4:**

Before deciding on further treatment, an MRI of the abdomen with contrast agent should be performed to better assess the extent of the venous thrombus. If the thrombus is still confined to the renal vein, a radical nephrectomy is the treatment of choice. In special situations (tumour-bearing single kidney, risk of dialysis) and a more peripheral tumour location and tumour thrombus without vessel wall infiltration, kidney preservation may even be possible (intraoperative decision, tumour thrombus mobilizable?, tumour completely resectable?). Cases in this regard have been described in the literature, but this should not be regarded as a standard procedure.

**LLM 1 (Chat GPT3.5):**

Treatment options for a patient with renal cell carcinoma presenting as a large mass at the lower pole of the kidney with a renal vein thrombus vary and depend on several factors, including the patient's overall health, tumor stage, and individual risk factors. Here are some possible treatment options:

1. surgical resection: for locally advanced renal cell carcinoma, as in this case, surgical removal of the tumor (nephrectomy) along with the renal vein thrombus is an important option. Depending on the exact location and extent of the tumor, a partial nephrectomy (removal of the tumor and part of the kidney) or a radical nephrectomy (complete removal of the kidney) can be performed.

2. thrombectomy: If the renal vein thrombus is extensive, a thrombectomy may be necessary. This involves removing the thrombus from the renal vein in order to restore normal blood flow.

3. targeted therapy: If surgical removal is not possible or the tumor is inoperable, targeted therapies can be used. These therapies target specific molecular changes in the tumor and can suppress tumor growth. Examples of targeted therapy options are tyrosine kinase inhibitors such as sunitinib, pazopanib or cabozantinib.

4. immunotherapy: Immunotherapeutic approaches, such as checkpoint inhibitors (e.g. nivolumab or pembrolizumab), can stimulate the patient's immune system to attack the tumor.

5. combination therapies: In some cases, combinations of targeted therapies or immunotherapy options may be considered to increase efficacy.

6. clinical trials: Depending on the patient's individual circumstances, participation in clinical trials for novel therapeutic approaches may be an option.

The final treatment decision should be made in close collaboration between the patient, the oncologist and the surgeon, taking into account individual health status, tumor characteristics and other relevant factors.

**LLM 2 (Chat GPT4.0):**

I can share some general information about treatment options for renal cell carcinoma that may be relevant to this case. However, it is important that this patient consults his doctor to discuss the best possible treatment strategy based on his specific situation, general health and other factors.

For a large renal cell carcinoma (15 cm in the case described) with renal vein involvement and potential lymph node involvement, but without proven organ metastases, the treatment options could be as follows:

1. surgical removal of the tumor:

- Radical nephrectomy: This involves removal of the entire kidney, surrounding fatty tissue, adrenal gland and nearby lymph nodes. In this case, removal of the renal vein thrombus may also be necessary.

- Partial nephrectomy: This is a kidney-preserving operation in which only the tumor and a small margin of surrounding healthy tissue are removed. With a large tumor, as in the case described, this option is probably less likely.

2 Local treatment options:

- Embolization of the renal artery: this is a procedure in which the blood supply to the tumor is blocked to reduce its size. This can be performed prior to surgery to facilitate the procedure or as a stand-alone therapy.

3. systemic therapies:

- Targeted therapy: these drugs, such as sunitinib or pazopanib, block specific pathways that promote tumor growth.

- Immunotherapy: For example, with checkpoint inhibitors such as nivolumab or pembrolizumab, which activate the immune system to attack the cancer.

4. adjuvant therapy:

- After surgical removal of the tumor, adjuvant therapy may be considered to reduce the risk of recurrence. This could include targeted therapy or immunotherapy.

5. neoadjuvant therapy:

- This refers to the use of systemic therapy options prior to surgical removal of the tumor to reduce its size and facilitate surgery.

It is important to emphasize that the choice of therapy depends on many factors, including the overall health of the patient, the accurate staging classification of the tumor, and the risks and benefits of each therapeutic option.

The management of patients with renal cell carcinoma is usually multidisciplinary, with urologists, oncologists, radiologists and other specialists working closely together to develop the best treatment plan.

The patient should ensure that they discuss all options and potential side effects with their treatment team and seek a second opinion if necessary.

It is essential that the patient discusses all treatment options with their doctor to find the most appropriate therapy for their situation. The above information is general and may have changed since my last update in 2021, so consultation with a specialist is essential.

**LLM 3 (Bard):**

The treatment options for a 55-year-old patient with renal cell carcinoma, which appears on CT as a 15 cm large mass at the lower renal pole with an additional renal vein thrombus, are as follows:

- Surgery: Surgery is the standard treatment for renal cell carcinoma. During surgery, the affected kidney is removed. If surgery is possible, it is the best way to stop tumor growth and cure the cancer.

- Chemotherapy: Chemotherapy can be used to treat renal cell carcinoma that cannot be operated on or that has returned after surgery. Chemotherapy can kill the cancer cells and stop tumor growth.

- Radiotherapy: Radiotherapy can be used to treat renal cell carcinoma that cannot be operated on or that has returned after surgery. Radiation therapy can kill the cancer cells and stop tumor growth.

- Immunotherapy: Immunotherapy is a new treatment option for renal cell carcinoma. Immunotherapy can stimulate the body's immune system to attack and kill the cancer cells.

The best treatment option for a 55-year-old patient with renal cell carcinoma is determined by a doctor based on the individual tumor stage, age and health of the patient

**Case 2**

A 57-year-old patient presents with severe right-sided colic pain in the flank. Imaging diagnostics show a ureteral stone. In the presence of which factors is a spontaneous passage not a valid option?

**Urologist 1:**

Spontaneous passage should not be awaited if it is unlikely (stone size >7mm), the patient is not pain-compensated despite extensive i.v. analgesia or complicating factors exist or occur during the course (including infected hydronephrosis, postrenal renal failure, fornix rupture).

**Urologist 2:**

If an infected urinary obstruction is suspected as a result of the outflow obstruction, urinary drainage must be restored immediately by placement of a urinary stent or nephrostomy insertion. Urinary diversion should also be performed in the event of fornix rupture or pain that cannot be treated with medication. Ureteral stones >6mm, especially if located in the proximal ureter, the probability of spontaneous passage is low, so that a ureteral stent insertion is advisable. Depending on the size and location of the stone, primary URS or ESWL can be discussed. Alkalinization of the urine is recommended for uric acid stones.

**Urologist 3:**If the size is not capable for spontaneous passage (probability is significantly reduced from >5mm), acute renal failure, increased infection parameters (infected hydronephrosis), non-cupable pain or clear fornix rupture, spontaneous passage should not be awaited.

**Urologist 4:**Spontaneous passage should not be awaited in the presence of infection, elevation of inflammatory parameters such as C-reactive protein and leukocytosis, and acute renal failure with creatinine elevation. If the stone size is >7 mm, spontaneous passage is not to be expected. In the case of analgesic-refractory colic and anatomical anomalies, it is also not advisable to wait for spontaneous passage. Furthermore, intervention should also be carried out in the event of a fornix rupture or persistent obstruction.

**LLM 1 (Chat GPT 3.5):**A ureteral stone that causes severe right-sided colic pain in the flank can be extremely painful. In some cases, the stone may pass spontaneously, while in other cases interventional treatment may be necessary. There are certain factors in which spontaneous drainage should not be waited for and immediate medical intervention is required. These include

1. size of the stone: larger stones are less likely to pass spontaneously. If the stone is about 6 mm or more in diameter, there is an increased risk of complications and blockages.

2. persistent symptoms: If pain persists or worsens despite adequate pain control and hydration, this could indicate a blockage or other complications.

3. infection or fever: The presence of infection or fever may indicate a urinary tract infection or a complication of ureteral calculi that requires immediate treatment.

4. unilateral renal dysfunction: if the stone affects kidney function on one side and causes congestion, this may lead to deterioration of kidney function that requires prompt treatment.

5. signs of renal failure: If the patient shows signs of acute renal failure, such as severe fluid retention, confusion or changes in consciousness, immediate medical attention is required.

6. severe urinary obstruction or blockage: If the stone completely blocks the flow of urine or causes severe urinary obstruction, this can lead to significant damage to the kidney and requires immediate relief.

7. Pregnancy: Special care should be taken with pregnant women as complications can occur during pregnancy that require prompt intervention.

It is important that a physician evaluates the patient's individual situation and recommends an appropriate treatment option based on factors such as stone size, location, symptoms and potential complications. In some cases, interventional treatment such as extraction of the stone using endoscopic methods or ESWL (extracorporeal shock wave lithotripsy) may be required to avoid complications and improve the patient's condition.

**LLM 2 (Chat GPT 4.0)**A ureteral stone can cause severe pain and, in many cases, pass spontaneously. However, there are certain situations in which waiting for the stone to pass spontaneously is not advisable and active treatment should be considered. These factors include

1. size of the stone: stones larger than 6-7 mm are less likely to pass spontaneously.

2. signs of infection: fever and/or signs of urosepsis (an infection that has spread to the blood) are absolute indications for immediate intervention.

3. unilateral kidney function or single kidney: patients with only one functioning kidney are at higher risk of kidney failure due to a blocked ureter.

4. uncontrolled pain: If the pain cannot be adequately controlled with analgesics, intervention should be considered.

5. persistent renal dysfunction: a blocked kidney can lead to renal dysfunction. If renal function does not improve, intervention may be required.

6. persistent obstruction: If the stone remains stuck in the ureter for more than 4-6 weeks, removal should be considered to minimize the risk of complications.

7. patients with a high occupational or social risk of waiting: for example, pilots, patients living alone in remote areas, or patients whose occupation requires constant concentration and where sudden colic could be catastrophic.

8. other medical conditions: for example, patients with only one kidney, patients with known renal dysfunction or patients for whom the use of NSAIDs (non-steroidal anti-inflammatory drugs often used to treat stone pain) is contraindicated.

It is important that the treatment decision is made in close consultation between doctor and patient and that the patient's individual risk factors and circumstances are taken into account.

Treatment options for ureteral stones that do not pass spontaneously include extracorporeal shock wave lithotripsy (ESWL), ureteroscopy with stone extraction or laser lithotripsy and percutaneous nephrolithotomy (PCNL).

In all cases, a patient with a ureteral stone should be treated under the care of a urologist or other qualified healthcare professional. It is important that the patient is closely monitored in order to detect complications and treat them at an early stage if necessary.

**LLM 3 (Bard)**
Spontaneous passage of a ureteral stone is the most common form of treatment. However, there are some factors where spontaneous passage should not be waited for. These include

- Pain that does not respond to painkillers

- Fever or chills

- Nausea and vomiting

- Blood in the urine

- Renal insufficiency

- A urinary tract infection

- Urinary retention

- Pregnancy

- A history of ureteral stones

If any of these factors are present, the ureteral stone should be removed as soon as possible. This can be done by surgery or non-surgical therapy such as ultrasound waves or shock wave therapy.

The best treatment method for a ureteral stone is determined individually by the doctor.
